# Exploring eco-anxiety among women amid climate-induced heat: a comprehensive review

**DOI:** 10.3389/fpsyg.2025.1480337

**Published:** 2025-06-19

**Authors:** Gayathri K. G, Vijayalakshmi P, Krishnan S, Rekha S, Latha P. K, Vidhya Venugopal

**Affiliations:** ^1^Department of Environmental Health Engineering, Sri Ramachandra Institute of Higher Education and Research, Chennai, India; ^2^Faculty of Allied Health Sciences, Sri Ramachandra Institute of Higher Education and Research, Chennai, India; ^3^Department of Physiology, All India Institute of Medical Sciences, Guwahati, Assam, India

**Keywords:** climate change, heat stress, women, eco-anxiety, psychological health

## Abstract

Eco-anxiety, the chronic fear of environmental doom, has become more frequent as climate change accelerates, particularly among disadvantaged population. This comprehensive review explores the relationship between eco-anxiety and gender differences, with a particular emphasis on women who experience stress and summarises the existing literature on the psychological and emotional responses to heat-related climate stressors. The current review critically examined a total of 21 articles and synthesised the scholarly literature on eco-anxiety, then it was reported using the Preferred Reporting Items for Systematic Reviews and Meta-Analyses (PRISMA-ScR). Women often face socioeconomic and physiological challenges, leading them to be more vulnerable to the effects of climate change, such as extreme heat events. This review was mainly focussed on the climate induced pathways to psychological consequences and gender differences with respect to eco-anxiety which strives to reveal targeted support systems and promote more inclusive climate resilience planning by comprehending the gender-specific dimensions of eco-anxiety. We observed that most of the evidence were from Western countries, hence global research is essential. According to our review, further study is needed to define eco-anxiety with respect to climate induced heat.

## 1 Introduction

Climate change (CC) is a global phenomenon affecting all regions of the world. According to 2009 Lancet Commission, the most significant worldwide health concern of the 21st century is the changes in the climate. The effects of global climatic changes, including heat waves, wildfires, hurricanes, flooding, and drought, are widely recognized as a potential threat to humankind (Majumder et al., 2023). It impacts a range of sustainable developmental issues including health, food security, employment, earnings, livelihoods, gender equality, education, housing, and poverty (Lundgren et al., 2013). In this case, extreme heat is one of the major CC issues, particularly affecting the poor. High temperatures have resulted in higher mortality in the past 15 years (Akhtar, 2020; Azhar et al., 2014; Michal Freedman et al., 2015). The duration and intensity of heat waves, as well as the average yearly temperatures are projected to increase globally (Domeisen et al., 2022).

CC has a substantial effect on individual’s physical health by increasing the incidence of heat-related illnesses (HRIs) (Lundgren et al., 2013). Although the evidence has shown that responses to climate-related events are associated with negative consequences for psychological health, such as elevated levels of stress, anxiety, and depression (Reser et al., 2011). These psychological issues are expected to increase in frequency as the environment continues to deteriorate (Clayton, 2020). The effects of CC on social and psychological well-being can be intensified by awareness and comprehension of it as a global environmental danger, which may have lasting impacts on psychological health. This link underscores how crucial it is to address climate change as a mental health problem as well as an environmental concern (Fritze et al., 2008).

One of the most increasingly concerning issues is the climate induced eco-anxiety (chronic fear of environmental doom) (Foster, 2022) and environmental deterioration, a comparatively unique phenomenon that is becoming more prominent day-by-day (Clayton et al., 2023) can be described as discomfort that stems from the perceived threat of ecological disasters, making the individuals feel helpless, anxious or sad (Reyes et al., 2023). This makes people feel helpless and prevents them from taking environmentally friendly actions because they think their actions will not make a difference in the bigger picture of cc (Pihkala, 2020). Many studies have reported that hotter months increase violence and criminal activity, linking aggressive attitudes to temperature (Miles-Novelo and Anderson, 2019). Climate-related anxiety can impact individual’s psychological well-being impacting sleep patterns, social impacts and economic loss (Lawrance et al., 2022). Moreover, multiple studies have reported a correlation between heat waves and their impacts on mental health and socio-behavioral changes among adults (Cianconi et al., 2020).

CC threatens women, especially the impoverished, who depend on natural resources for their livelihoods. Women have important roles in CC adaptation and protection for their families, but they have less decision-making power (Koggel and Bisman, 2017). Women are more vulnerable to CC due to economic, cultural, and social challenges that differ across regions. They have less access to CC adaptation resources such as land, finance, agricultural inputs, decision-making authority, technology, training, and extension services than men, especially in low-resource settings (Aggarwal et al., 2018). They also provide their families with water, energy for cooking and heating, and food security, but women lack authority over environmental goods and services, and benefit distribution. Women labour twice as hard for their families during droughts, heat extremes, and floods, leaving less time to learn, earn, and adapt to CC. Climate conflict and natural resource competition-related domestic and communal violence leave women psychologically exhausted and anxious (Campbell, 2013).

Given the relegation of women to subordinate backgrounds, they are at optimum risk of the psychological consequences of CC which include, but are not limited to depression, suicide, Post-Traumatic Stress Disorder (PTSD), neuropsychiatric symptoms arising from environmental impacts (Rothschild and Haase, 2023). Studies show that women are much more inclined to experience additional emerging mental health issue – eco-anxiety – within the context of CC. Climate anxiety due to heat stress are some of the severe challenges that women are facing in their lifetime across the global world especially in Africa, Europe, Bangladesh, Philippines and India (Goudet et al., 2023). South Indian rural communities, which depend on agriculture and natural resources, are especially vulnerable to CC (Sridevi et al., 2014). In these areas, women face climate-related issues due to their involvement in agriculture, household administration, and caregiving (Heeren et al., 2022). Many studies indicated that due to heat, farmers especially, pregnant women in Gambia get exposed to heat stress during work with many health complains and may also be having other mental health effects (Heeren et al., 2022; Bonell et al., 2021; Boyd et al., 2024).

Climate anxiety, a continual fear of environmental disaster, affects women’s psychology and health (Clayton et al., 2023; Heeren et al., 2022). It can be exacerbated by overexposure to CC in the media, discussions about CC, and the perception that the damage will soon affect them and their families (Otto et al., 2017). However, the psychological consequences of heat extremes on women are receiving little attention in public and scientific debate when compared to the physical effects (Fritze et al., 2008). Although there is ample literature in community resilience, particularly the practice and impact of the ability of women to cope with ecological disasters, there is still limited information about females with eco-anxiety. In the changing environment, it is critical to understand how warming affects women’s mental health in both direct and indirect ways. This review attempts to address this by providing a synthesis of the literature related to eco-anxiety and its impact on women who are susceptible to heat stress owing to CC.

## 2 Methodology

**Figure 1:**
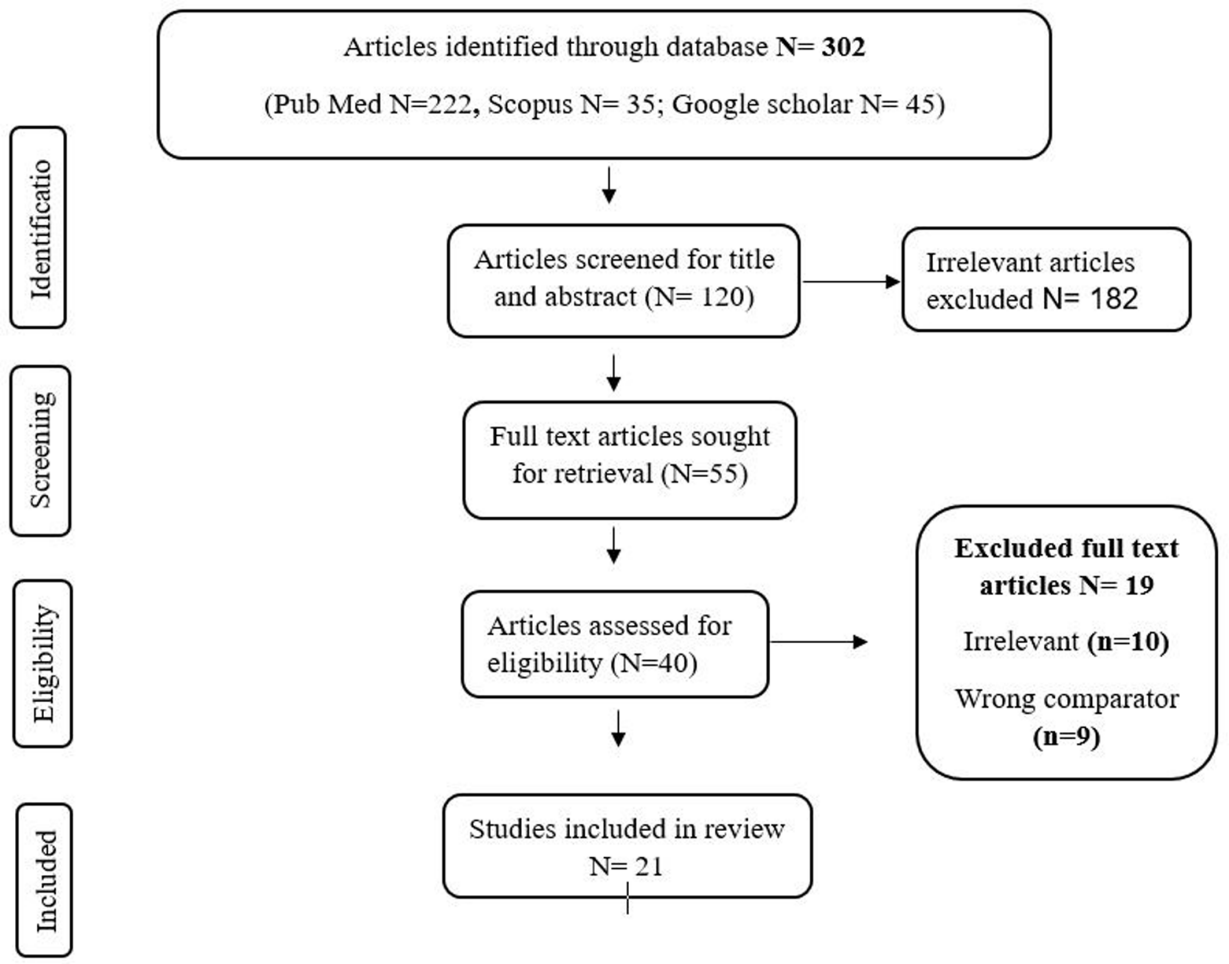
PRISMA flow diagram of the article’s selection process.

To examine the impact of heat stress on women’s psychological health (eco-anxiety), we conducted a comprehensive literature survey (Figure 1). Studies published between 2000 to 2024 were included, with a focus on empirical research, review articles, and grey literature relevant to this topic was searched in databases such as PubMed, Scopus, using keywords such as “climate change,” “climate anxiety,” “eco-anxiety,” “heat stress,” “women,” “mental health,” and “environmental stress.” To identify additional pertinent articles that were not included in the electronic database searches, a Google Scholar search was also implemented. This review addresses few research question such as what is the extent of the literature on eco-anxiety in women that is associated with climate-induced heat? In what ways does climate-induced heat exacerbate eco-anxiety in women? The studies were selected based on the selection criteria to ensure its relevance to research objectives.

### 2.1 Selection criteria

- Inclusion criteria for the literature encompassed peer-reviewed articles published within the last ~20 years, focusing specifically on climate change, climate change anxiety or eco-anxiety, and gender-specific impacts. Studies were included if they were empirical, reviews, case studies, or meta-analyses.
- Exclusion criteria filtered out non-English articles, those that focused solely on general climate change without addressing eco-anxiety or gender with respect to heat, and publications without accessible full texts.

### 2.2 Data extraction

To find relevant articles, we utilized the following search criteria: climate change AND climate change anxiety AND eco-anxiety AND women AND heat (OR climate induced heat OR high temperature OR hot temperature OR heat wave OR heat stress OR extreme temperature) (OR mental health OR psychological health OR mental wellbeing OR anxiety OR depression OR psychological distress OR emotional distress OR psychological impacts). Initial screening of titles and abstracts was performed to assess relevance. Full texts of appropriate articles were analysed in detail, and those meeting the inclusion criteria were chosen for data extraction. The extraction process focused on study characteristics, such as authors, publication year, and journal, along with population characteristics, methodological details, and key findings related to Climate anxiety, eco-anxiety and climate-induced heat, and gender-specific insights. This study investigates the impact of climate-induced heat on women’s eco-anxiety. We excluded research studies that did not include women or addressed concepts unrelated to heat stress and articles that were non-English.

### 2.3 Data synthesis

To understand the impact of climate-induced heat on eco-anxiety among women, we conducted a comprehensive review of existing literature. Our primary objective was to determine the impacts of climate-induced extreme temperatures and the associated psychological impacts. The studies included in this review were selected based on the **PICO**: Population; Intervention; Comparator; and Outcomes. Women were the target population, particularly those who are primary caregivers or economically vulnerable, and exposed to climate-induced heat waves; exposures to higher temperatures as the intervention; Comparing women’s experiences of eco-anxiety with those of other populations; and Outcome: The impact of interventions on reducing eco-anxiety and improving mental health, resilience, and adaptive capacity among women. Understanding these dimensions through the PICO framework can help develop targeted strategies that support women’s mental well-being amid escalating climate threats.

Initial searches yielded a total of 302 citations (Figure 1), and encompassed a range of study designs, including qualitative interviews, quantitative surveys, and mixed methods approaches. Key themes emerging from the literature included which then exported to Zotero for analysis. Title and abstract screening were performed on the unique articles (*n* = 302). Many of the irrelevant publications (*n* = 182) were excluded at this stage. Full-text analysis was performed for the remaining publications (*n* = 55). The studies were selected using the Preferred Reporting Items for Systematic Reviews and Meta-Analyses (PRISMA) criteria. PRISMA criteria was selected instead of traditional systematic review approaches to ensure methodological rigor and transparency in examining the existing evidence. This made it possible to consider the entire spectrum of eco-anxiety with its intersection with climate change, climate-induced heat, and unique vulnerabilities experienced by women. By adopting this PRISMA-ScR, we were able to review a wide range of study designs, including qualitative, quantitative, and mixed-methods research, which is critical for understanding the multidimensional nature of eco-anxiety. PRISMA-ScR maps evidence sources utilizing multiple study methods and identifies the gap in research on gendered eco-anxiety. The depth of analysis is limited by its capacity to thoroughly evaluate study quality. This review synthesise narratives but not evidence for meta-analyses. After removal of irrelevant and wrong comparator articles, a total of 21 articles were selected for review. The paper’s title, abstract, and full-text content were reviewed first. We recognize the potential for selection biases in the inclusion of these articles. One such bias may arise from the dominance of Western-centric research, which limits the representation of studies from low- and middle-income countries (LMICs), particularly in tropical and climate-vulnerable regions.

## 3 Results and discussion

The studies summarised in this review (Table 1) shows substantial evidence that eco-anxiety is a growing concern among all populations, with a particular emphasis on women, who are disproportionately affected by CC-induced stressors, like heat waves. This section delves into the existing literatures, with an emphasis on the distinctive vulnerabilities and experiences of women who are confronted with eco-anxiety in the context of climate-induced heat.

**Table 1:** Summary of the literatures on heat, eco-anxiety and mental health.

S. No.	Author	Year	Location	Title of the study	Purpose of the study	Study design	Sample population	Sample size	Key findings
1.	Agoston C et al	2022	Hungary	Identifying Types of Eco-Anxiety, Eco-Guilt, Eco-Grief, and Eco-Coping in a Climate-Sensitive Population: A Qualitative Study.	To understand the psychological effects of climate change, focusing on eco-anxiety, eco-guilt, eco-grief, and eco-coping mechanisms within a climate-sensitive population.	Qualitative study	Adults	17 (6 male, 11 female)	Participants experienced various symptoms of eco-anxiety, including worry, helplessness, and anxiety about the future. The study found that eco-anxiety could cause temporary functional impairment, similar to general anxiety disorders.
2.	Alexander Savu et al	2022	UK	Temperature highs, climate change salience, and Eco-anxiety: early evidence from the 2022 United Kingdom heatwave.	The purpose of the study was to assess the impact of an unprecedented mid-July 2022 heatwave in the United Kingdom on climate change salience and people’s well-being, focusing on the role of climate anxiety (CA).	Observational study	Adults	The sample size is not explicitly mentioned	The study concluded that extreme weather events like the 2022 heatwave exacerbate climate anxiety, making climate change more salient to the public and affecting overall well-being
3.	Anne Weaver et al	2022	North Caroline	Is short-term exposure to heat associated with mental health compared to other conditions	This study examined associations between short-term (5-day) apparent temperature and health visits for anxiety and/or depression in North Carolina, using electronic health records from a random sample of adults.	Random sampling	Adults	17,145	Findings revealed that individuals with anxiety and depression were younger, more likely to be female compared to those with other diagnoses. The prevalence of a diagnosis of anxiety and/or depression was slightly higher with an increase in the five-day mean apparent temperature,
4.	Boyd et al	2024	Australia	Eco-anxiety among regional Australian youth with mental health problems: A qualitative study	The present study purpose was to address gap by focussing on the lived experiences of regional Australian youth with recent experience of climate related disasters alongside clinical insights from those involved in their care.	Qualitative study	Australian youth	25	Three dimensions of eco-anxiety were identified by clinicians and client’s helplessness in the present, hopelessness about the future and acute stress and anxiety related to experiences of severe flooding and fires.
5.	Bundo et al	2021	Switzerland	Ambient temperature and mental health hospitalizations in Bern, Switzerland: A 45 year time-series study	The purpose of the study was to investigate the short-term association between ambient temperature and mental health hospitalizations in Bern, Switzerland	Time series	Adults	88,996	The association between ambient temperature and mental health hospitalizations was found to be linear, similar to studies conducted in Toronto and Hong Kong, with no evidence of larger risks during warmer temperatures or heatwaves
6.	Chan et al	2018	Hong kong	Association between Ambient temperatures and Mental Disorder Hospitalizations in a Subtropical City: A Time-Series Study of Hong Kong Special Administrative region	The aim of the study was to investigate the association between ambient temperatures and mental disorder hospitalizations in a subtropical city, specifically the Hong Kong Special Administrative Region.	Time series	Adults	44,600	The study revealed a positive association between ambient temperatures and mental disorder hospitalizations in Hong Kong, with temperature showing a significant impact on mental health outcomes
7.	Clayton et al	2023	USA	Psychological and Emotional Responses to Climate Change among Young People Worldwide: Differences Associated with Gender, Age, and Country	The Purpose of the study was to investigate the psychological and emotional responses to climate change among young people aged 16–25 worldwide, focusing on differences associated with gender, age, and country of residence.	Cross-sectional design	Young people 16–25	10,000	**Gender Differences:** Female respondents reported higher levels of concern and negative emotions related to climate change compared to male respondents. Male respondents were more optimistic and exhibited greater faith in governmental responses to climate change.
8.	Feathers et al	2022	Australia and New Zealand	A psychometric evaluation of the Climate Change Anxiety Scale	The study aimed to evaluate the psychometric properties of the Climate Change Anxiety Scale (CCAS) developed by Clayton and Karazsia to measure anxiety related to perceptions of climate change	Conceptual replication	Residents	401	The CCAS showed weak-moderate positive correlations with ecocentrism, generalised anxiety, non-specific distress, and concern about climate change, and a weak negative correlation with climate change denial. Confirmatory factor analyses indicated that the two-factor model specified by Clayton and Karazsia had reasonable global fit, but neither a one- nor two-factor model met the preregistered criteria for a good fit. The CCAS demonstrated good to excellent internal consistency and good test–retest reliability
9.	Heeren A et al	2022	African European countries	On climate anxiety and the threat, it may pose to daily life functioning and adaptation: a study among European functioning and adaptation: a study among European and African French-speaking participants	The purpose of the study was to investigate the prevalence and variations of climate anxiety across different demographic groups (such as gender and age) and its associations with adaptive behaviors, specifically pro-environmental behaviors.	cross-sectional design	French-speaking population	2080 French-speaking participants,	11.64% of 2080 French-speaking participants from African and European countries reported frequent climate anxiety. 20.72% experienced daily life functional consequences due to climate anxiety. Women and younger individuals exhibited significantly higher levels of climate anxiety.
10.	Hogg et al	2021	Western countries	The Hogg Eco-Anxiety Scale: Development and Validation of a Multidimensional Scale	The purpose of the study was to develop and validate a new scale, the Hogg Eco-Anxiety Scale (HEAS-13), to measure eco-anxiety as a multidimensional construct.	Cross-sectional design	Students- Young adults	924	The study found that eco-anxiety is a multidimensional construct with four distinct dimensions: affective symptoms, ruminative thoughts, impairment to behavioral and social functioning, and anxiety about personal impact on the planet. Eco-anxiety was found to be stable over time for some dimensions, while others fluctuated in response to environmental stimuli.
11.	Innocenti et al	2023	Italy	How Does Climate Change Worry Influence the Relationship between Climate Change Anxiety and Eco-Paralysis? A Moderation Study	This study presents the validation of Italian versions of Hogg’s Eco-Anxiety Scale (HEAS) and the Eco-Paralysis Scale. It also investigates the effects of worry on eco-anxiety and eco-paralysis.	Quantitative study with moderation analysis	Italian individuals	150	Both HEAS and the Eco-Paralysis Scale showed good psychometric properties, suggesting a one-factor structure for each and high internal consistency. Moderating Role of Worry: Climate change worry acts as a moderator between climate change anxiety and eco-paralysis, influencing individuals’ ability to transform anxiety into action
12.	Innocenti et al	2023	Italy	How Can Climate Change Anxiety Induce Both Pro-Environmental Behaviors and Eco-Paralysis? The Mediating Role of General Self-Efficacy	The studies purpose was to investigate how climate change anxiety influences pro-environmental behaviors (PEBs) and eco-paralysis, focusing on the mediating role of general self-efficacy	cross-sectional design	Italian individuals	394	Climate change anxiety had a direct positive effect on encouraging PEBs and an indirect negative effect on PEBs through self-efficacy, potentially leading to eco-paralysis. The findings highlighted the importance of developing coping strategies such as PEBs to help individuals manage climate change anxiety effectively.
13.	Mercer JA et al	2022	USA	Children and Climate Anxiety: An Ecofeminist Practical Theological Perspective	The purpose of the study was to explore and understand children’s vulnerability to climate change and climate anxiety through the perspective of ecofeminist practical theology.	Qualitative and interpretive approach	children	NA	The study explores the intersection of children’s vulnerability to climate change and their experiences of climate anxiety. Ecofeminism, integrating feminist concerns with ecological issues, provides a lens to understand how children are disproportionately affected by climate-related distress.
14.	Li et al	2020	US	Temperature and self-reported mental health in the United States	The study aimed to estimate the association between temperature and self-reported mental health in the United States by analyzing individual-level mental health data	Retrospective	Adults	3 million	Cooler days were found to reduce the probability of reporting days of bad mental health, while hotter days increased this probability.
15.	Nori sarma et al	2022	US	Association Between Ambient Heat and Risk of Emergency Department Visits for Mental Health Among US Adults, 2010 to 2019	To investigate the association between ambient heat and mental health–related emergency department (ED) visits in the contiguous US among adults overall and among potentially sensitive subgroups	Case-crossover study	Adults	3,496,762	Extreme heat days are associated with increased rates of mental health–related ED visits. This information can aid clinicians in preparing for increased demand during periods of extreme heat.
16.	Niu et al	2023	New York	Temperature and mental health–related emergency department and hospital encounters among children, adolescents and young adults	To examine the association between high ambient temperature and acute mental health-related healthcare encounters in New York City for children, adolescents and young adults.	Case crossover study	Children, adolescents & young adults	82,982	Elevated ambient temperatures were associated with acute mental health ED or hospital encounters across childhood, adolescence and young adulthood.
17.	Reyes et al	2001	Philippines	An investigation into the relationship between climate change anxiety and mental health among Gen Z Filipinos	The purpose of the study was to investigate the link between climate change anxiety (or eco-anxiety) and mental health among young adults in the Philippines.	Cross-sectional design	Young adults 18 to 26 years	433 (145 male and 288 female)	The study found that climate change anxiety significantly predicts 13.5% of the variance in overall mental health, particularly impacting Psychological Distress but not Psychological Well-being.
18.	Schwartz et al	2022	United states	Climate change anxiety and mental health: Environmental activism as buffer	The purpose of the study was to Investigate the association between climate change anxiety (CCA) and symptoms of Major Depressive Disorder (MDD) and Generalised Anxiety Disorder (GAD) among emerging adults.	Mixed method design	Adults 18–35 yrs	284	Both subscales of CCA (cognitive emotional impairment and functional impairment) are linked to GAD symptoms. Only the functional impairment subscale is linked to higher MDD symptoms.
19.	Thomson et al	2023	Canada	The relationships among nature connectedness, climate anxiety, climate action, climate knowledge, and mental health	The purpose of this study is to examine how climate anxiety influences the above-described relationships.	Cross-sectional design	Adults	327	Individuals with higher scores reported significant psychological distress, demonstrating the scales’ effectiveness in capturing the intensity of eco-anxiety. The scales successfully predicted psychological distress and were useful for identifying individuals at risk for mental health issues due to climate anxiety. The scales revealed links between climate anxiety and pro-environmental behaviors, suggesting that anxiety might drive both individual and collective actions.
20.	Jang et al	2022	South Korea	Validation of the Climate Change Anxiety Scale for Korean Adults	The study aimed to translate the Climate Change Anxiety Scale (CCAS) into Korean and verify its reliability and validity	Methodological study	Korean adults 19 to 65 yrs	459	The study successfully translated the CCAS into Korean and verified its reliability and validity. The levels of climate change anxiety observed in this study were lower than in other nations, but the tool could still be used to monitor anxiety levels among South Koreans to help prevent mental health problems and improve psychological well-being
21.	Wahid et al	2023	Bangladesh	Climate-related shocks and other stressors associated with depression and anxiety in Bangladesh: a nationally representative panel study	The study aimed to analyze the associations between climate-related factors, sociodemographic variables, and the prevalence of depression and anxiety in Bangladesh	Stratified random sampling	Adults	3,606	An increase in mean temperature preceding the surveys was linked to higher odds of anxiety and co-occurring depression and anxiety, while humidity was associated with co-occurring depression and anxiety

### 3.1 Climate induced pathways to psychological consequences

The psychological health consequences of CC can range from mild stress and distress symptoms to severe illnesses, including sleeping difficulties, depression, PTSD, and suicidal ideation (American Psychological Association, ecoAmerica, 2017). Ágoston et al. (2022) found that many adults reported significant worry about CC, with experience of maladaptive eco-anxiety, which impaired daily functioning. Further effects may include the impact on individuals and communities in daily existence, beliefs, and their experiences, requiring the coping ability, understanding, and responding to CC and its consequences (Jang et al., 2023). Depression, PTSD, generalised anxiety disorder (GAD), rise in substance use or misuse, and suicidal thinking are common reactions to catastrophic events resulting in social life disturbance, such as the loss of life, resources, social support, and communication channels, or massive migration (Shah, 2020; Innocenti et al., 2023). A recent study (Reyes et al., 2023) found that CC anxiety predicts 13.5% of the variance in overall mental health among young Filipinos, particularly increasing psychological distress (Innocenti et al., 2023). Similarly, many other studies (Schwarz et al., 2022) also identified a strong association between CC anxiety and symptoms of GAD and major depressive disorder (MDD) among emerging adults in the United States, suggesting that eco-anxiety can exacerbate pre-existing mental health conditions (Schwartz et al., 2023).

Climate-induced heat stress affects psychological well-being and behavior, impacting mental health before and after extreme weather events (Clayton et al., 2021; Thomson and Roach, 2023). Extreme temperatures can result in a rise in hostility, aggressive thoughts, and potential behavior, as well as physical and psychological fatigue, mood disorders, and other related issues (Padhy et al., 2015). Prolonged exposure to high temperatures has been linked to increase irritability, and depressive symptoms (Rony and Alamgir, 2023). And weaver reported that most of the women were more likely diagnosed with anxiety and depression for per degree rise in temperature (Weaver et al., 2022). There is a notable increase in heat-related violence (Anderson and Bell, 2009) and a rise in suicide rates, particularly during the main summer season (Burke et al., 2019). These interrelated consequences highlight the wider significance of CC on psychological wellness.

Among 2,243,395 individuals, 56.8% of women and 43.2% of men visited emergency departments (ED) for substance use disorders (most prevalent) followed by anxiety, stress-related, and somatoform disorders, mood disorders. Along with those schizophrenia, schizotypal, and delusional disorders, self-harm, and childhood-onset behavioral disorders were also increased on days of extreme temperatures (Nori-Sarma et al., 2022). When the temperature is between 15 and 20°C, there is a lower likelihood of experiencing poor mental health, as the temperature gets hotter, the probability of reporting terrible mental health increases (Li et al., 2024; Li et al., 2020). The investigation done by Bundo et al. (2021) reported, the risk of hospitalisation increased by 4.0% for each 10°C increase in the mean daily temperature. These high ambient temperatures were linked to acute mental health hospital visits even among children, youth, and young adults (Niu et al., 2023). In a study of 3,606 adults, a 1°C rise in temperature was associated with increased odds of anxiety (6.0%), depression (16.3%), and co-occurring cases (4.8%) (Wahid et al., 2023).

**Figure 2:**
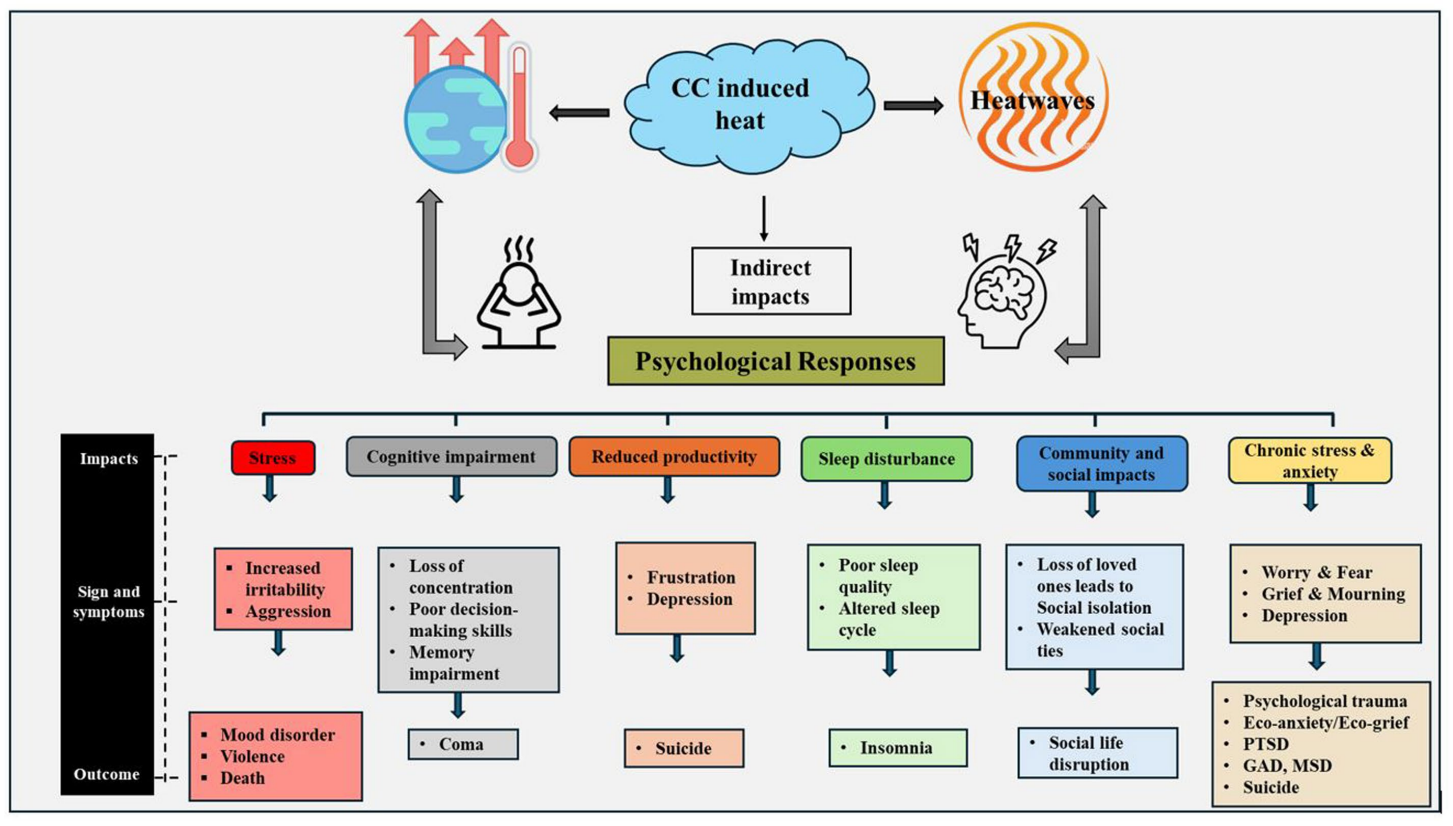
Climate change induced heat and its physical and psychological impacts on health.

Extreme heat was found to have both direct and indirect effects on psychological health (WHO, 2024) which leads to the chronic effects which causes psychological issues like stress, anxiety and depression (Thomson and Roach, 2023). As awareness on global warming and ecological degradation grows, terms like “climate anxiety,” and “eco-anxiety” enter our vocabularies, describing the impact of climate change on human mental health and wellbeing. Distress over climate change disproportionately impacts children, who also are more susceptible to the broader health, economic, and social effects brought about by environmental harm. In this paper, I also explored on children’s vulnerability to climate change and climate anxiety through the lens of ecofeminist practical theology. Ecofeminism brings the liberator concerns of feminist theologies into engagement with those theologies focused on the life of the planet (Mercer, 2022). Drawing on ecofeminism, practical theology must continue and deepen its own ecological conversion, and practical theologies of childhood must take seriously the work of making an ecological home, oikos, in which children are embedded as a part of the wider ecology that includes the more-than-human world. This requires foregrounding religious education with children toward the inhabitants of the earth in good and just ways. Figure 2 provides a framework of climate induced heat and its associated psychological responses on human well-being. These research links high heat events to anxiety, sadness, stress, and climate anxieties, hurting mental health and emphasising the need for preparedness and resilience (Atiqul Haq et al., 2024).

### 3.2 Gender differences in eco-anxiety

Women and men experience CC differently, as gender inequalities persist around the world, affecting the ability of individuals and communities to adapt. Climate-induced heat has a significant impact on women’s health and well-being, as their anatomy, physically demanding work, and limited access to sanitation facilities make them particularly vulnerable to heat stress (Amoadu et al., 2023). Prolonged exposure to high temperatures increases women’s risk of heat stress, dehydration, and chronic illnesses such as cardiovascular and respiratory conditions, especially for those in agriculture and informal sectors with limited cooling access (Venugopal et al., 2016). Acute impacts include heat exhaustion, heatstroke, and psychological distress, with marginalized women facing higher risks of anxiety and depression due to compounded effects of heat stress and socio-economic vulnerabilities (Clayton, 2020; Thomson and Roach, 2023). Additionally, factors like limited infrastructure, lower socio-economic status (SES), frequent engagement in agriculture and natural resource management, and household sustenance make them more susceptible to psychological impacts. Women, youth, and low-income people are more susceptible to disaster-related anxiety and mood disorders (Venugopal et al., 2016) due to poverty and a lack of resources. Women in low SES have limited access to cooling interventions at home or at their workplaces, as well as information and education about climate change and its impacts. This lack of awareness hinders their ability to adapt to changing conditions and exacerbates the psychological stress associated with uncertainty, which can lead to health risks, dehydration, and mental distress (Amoadu et al., 2023). Additionally, a study demonstrates that short-term exposure to high temperatures may increase the risk of anxiety and depression in women (69.7%) due to the psychological distress these extreme events cause, underscoring the significance of taking climate factors into account in mental health studies (Fang et al., 2023).

Evidence from the European heatwave shows higher mortality among women, mainly due to greater life expectancy and elderly vulnerability. Younger women also face risks from occupational exposure, caregiving roles, and limited access to cooling and healthcare (Fang et al., 2023). Mercer (2022) and Boyd et al. (2024) underscore the importance of understanding the unique vulnerabilities and experiences of women in the context of climate anxiety. Alexandru Savu’s (Vésier and Urban, 2023) research on women during the heat wave of 2022 in Britain revealed that extreme heat events escalate climate anxiety, thus intensifying eco-anxiety. According to Heeren et al. (2022), people living in France had reported higher climate anxiety than other countries, with women and youngsters scoring significantly higher. Clayton et al. (2023) also detected a higher prevalence of worry and distress associated with CC in women than in men, indicating a gender-based risk for developing eco-anxiety. The above disparities underpin the need for gender-sensitive interventions in addressing eco-anxiety. Young people and women, with first-hand experience of climate incidents bear higher levels of eco-anxiety and climate distress because they shape their future decisions (Savu, 2023). This implies that climate change and heat psychologically affect women by altering their educational endeavours, financial planning, and even family planning. The intersectional approach highlighted in Mercer (2022) and Boyd et al. (2024) works on the hypersensitivity of climate change to affect the vulnerable. This calls for a combination of eco-feminism and an intersectional analysis of the effects and management of eco-anxiety, with a significant emphasis on the support of the affected vulnerable groups.

The reviewed research reveals a lack of adequate literature, underscoring the urgent need for not only climate awareness but also informed behavioral patterns. These should be equitable, considering gender differences and the marginalisation of specific demographics. Additionally, they should emphasise the creation of the necessary capacities to prevent the adverse effects of climate-related stressors on mental health.

## 4 Research gaps and future needs

### 4.1 Research gaps

Despite strong evidence of eco-anxiety prevalence among women, there exists several research gaps. Further, longitudinal studies are needed to assess eco-anxiety over time among women. Most research is conducted on western populations, necessitating studies with broad, global approach, with more studies in tropical settings and climate-vulnerable Low and Middle Income Countries. Additionally, there is a scarcity in literatures on the impact of eco-anxiety with respect to climate induced heat among women, which should be investigated further. Further research is needed on this interdisciplinary approach, as well as the role of policy and advocacy in reducing the levels of eco-anxiety. Addressing these gaps will help us better understand and manage eco-anxiety.

### 4.2 Future needs

Given the substantial evidence of eco-anxiety, particularly among women disproportionately affected by CC-induced heat, several future needs are crucial. Strategies for mental health must incorporate climate awareness and establish support systems that focus on resilience and coping mechanisms. Gender-sensitive approaches are essential, addressing women’s specific vulnerabilities and promoting gender equality in climate action planning. To develop targeted interventions, longitudinal studies and data collection on vulnerable populations using validated scales specific to regions are necessary (Arezki et al., 2018; Feather and Williams, 2022). Policy efforts must integrate mental health considerations into CC plans, securing funding for mental health initiatives. Educational campaigns should raise awareness about climate change’s psychological impacts and incorporate climate education into school curricula. Support networks and community programmes should focus on building social cohesion and resilience, providing safe spaces for sharing experiences and coping strategies. We can develop a more inclusive and resilient approach to addressing these needs to mitigate the psychological consequences of CC-induced heat.

## 5 Recommendations

To successfully address the increased eco-anxiety experienced by women because of climate change, comprehensive mental health support services must be prioritised. This includes providing accessible psychological services tailored to women’s individual needs, particularly in vulnerable populations such as rural areas and places prone to climate-related disasters. Integrating mental health into primary care settings and conducting community outreach programmes to increase their awareness can assist to reduce the psychological impact of eco-anxiety. In addition, providing culturally sensitive and gender-responsive mental health care is essential, as women in regions around the world might have different coping strategies and support systems.

Interventions tailored to gender and age groups must also be developed and tested. Pregnant women and mothers should receive special attention, as they can develop increased eco-anxiety with concerns regarding the future of their children in a new climate. Mental health initiatives based on workplaces can also be useful, particularly for women who are employed in climate-sensitive jobs like agriculture and outdoor work. Conducting workshops on coping strategies like mindfulness, deep breathing or meditation can further aid in managing eco-anxiety. Workplace initiatives, including mental health screening, flexible working conditions, and cooling interventions, are essential for women working in climate-sensitive jobs. Furthermore, boosting awareness of psychological issues related to climate change through education and public campaigns is critical. Climate mental health literacy should be incorporated in school education and public campaigns to fight stigma and enhance resilience. To safeguard health from severe temperatures, targeted public health monitoring, air conditioning expansion, and “urban heat island” mitigation should be planned (Hogg et al., 2021). These efforts should strive to reduce the stigma associated with getting help for eco-anxiety while also providing practical coping skills. Furthermore, developing support networks and peer groups in which women can share their concerns and experiences might help to build resilience and improve psychological well-being in the face of climate-related uncertainties. By addressing these issues holistically, society can better assist women in navigating and adjusting to the psychological obstacles offered by CC. Given that women are more likely to experience higher levels of eco-anxiety, tailored interventions are necessary to address their specific needs.

## 6 Strengths

- Comprehensive summary of available literature examines the prevalence and effects of eco-anxiety on women.
- Highlights research gaps, including the need for longitudinal studies and global perspectives.
- Proposes practical policy initiatives and mental health assistance for women.
- Advocates for community support networks.
- Demonstrates a comprehensive understanding of eco-anxiety as a socio-psychological disorder exacerbated by climate change.
- Utilized the covidence tool to streamline article selection and data extraction, ensuring methodological rigor and minimizing bias in the review process.

### 6.1 Limitations

- Relies on Western research, limiting its applicability to other cultural contexts which affects the generalizability of the findings to diverse cultural, socioeconomic, and geographical contexts, particularly in low- and middle-income countries (LMICs).
- Lacks studies conducted in India, especially concerning climate-induced heat among women. The existing body of literature on eco-anxiety and climate-induced heat largely stems from high-income countries, where socio-economic conditions, gender dynamics, and healthcare systems differ significantly from those in LMICs. As a result, the psychological impacts of climate change on women in vulnerable regions, where heat exposure and resource constraints are more pronounced, may be underestimated or inadequately represented.
- Variation in methodologies across studies affects comparability. This review differ in topic and scientific quality, with some being descriptive and others empirical and did not assess study quality or exclude studies on the basis of potential bias.
- Makes policy recommendations but does not address challenges in implementing gender-sensitive policies in diverse socio-political contexts.

## 7 Conclusion

The present literature review gives an understanding of the extent of climate related eco-anxiety. Thus, the review helps to advance knowledge about the social aspects of CC adaptation and resilience in deprived populations. Therefore, this review shows that eco-anxiety is a massive problem for women in line with the existing data of affected women, intensified by heat caused by climate change. Women face unique challenges and vulnerabilities that heighten their anxiety about environmental changes. While community engagement and mental health interventions offer some relief, there is a critical need for gender-sensitive policies and further research to address this growing concern. Future research that addresses gaps in the literature and focus on women’s specific experiences can contribute to a better understanding of eco-anxiety and assist develop effective methods to support women’s mental health in the face of climate change. This also provides a comprehensive overview of the current state of research on eco-anxiety in women amid climate-induced heat, highlighting key findings, gaps, and recommendations for future research and policy interventions.
